# Longitudinal Impact of an Interdisciplinary Course on Aging for First-Year Students

**DOI:** 10.1093/geroni/igaa057.1437

**Published:** 2020-12-16

**Authors:** Matthew Picchiello, Nancy Morrow-Howell, Susan Stark, Brian Carpenter

**Affiliations:** 1 Washington University in St. Louis, University City, Missouri, United States; 2 Washington University in St. Louis, St. Louis, Missouri, United States

## Abstract

Undergraduate courses on aging have the potential to counteract negative stereotypes about older adults and to shift students’ academic plans as they learn about aging-related opportunities. For six years we have taught an interdisciplinary course on aging for first-year undergraduate students. We present longitudinal data on students’ attitudes and academic trajectories after taking the course. Students who took the course (n = 314) and comparable students who were not in the course (n = 353) were surveyed prior to and at the end of their first semester and at the end of each subsequent academic year. At each time point students rated the degree to which aging issues are relevant to their personal and professional lives. Students also reported aging-related curricular and extracurricular activities they pursued. Multivariate repeated-measures analyses revealed a significant interaction such that personal and professional relevance of aging issues were lower and remained stable for students not in the class, and were higher and increased for students in the class, F(2,226) = 13.18, F(2,226) = 14.94, p’s < .01. However, for course students, relevance returned to baseline levels by the end of their first year and remained constant in subsequent years. Results from chi-square analyses revealed that students in the class reported more engagement in aging-related courses, χ2(1) = 8.3, research projects, χ2(1) = 90.1, and extracurriculars, χ2(2) = 20.6, p’s < .01. Results suggest that exposing students to information about aging early has the potential to alter academic trajectories, highlighting the importance of early education.

